# Echographic Evaluation of a Subconjunctival Cystic Lesion

**DOI:** 10.1155/2017/5401850

**Published:** 2017-04-12

**Authors:** Maddalena De Bernardo, Gennarfrancesco Iaccarino, Valeria Russo, Nicola Rosa

**Affiliations:** ^1^Department of Medicine and Surgery, University of Salerno, Salerno, Italy; ^2^2nd University of Naples, Naples, Italy

## Abstract

Migration of intraocular silicone oil, used in the treatment of complicated retinal detachment, has been rarely described, but when it happens it can arise with a differential diagnosis with scleral buckling extrusion, tumor, dermoid, ocular cysticercosis, and abscess. The presence of silicone oil in the eye gives very ugly echographic pictures, but these kinds of pictures can be very useful in making a differential diagnosis in the above-mentioned cases. A 39-year-old white female complained of the presence of conjunctival hyperemia and tearing in the right eye (RE); her visual acuity was hand motion, and the intraocular pressure was 14 mmHg. In the upper nasal quadrant a dome shaped lesion was detected. Due to the lens opacities, the patient underwent an echographic examination, which revealed the presence of silicon oil both in the vitreous chamber and in a large subconjunctival space, corresponding to the lesion. This article in addition provides a possible explanation of such cystic formation and discusses the risk factors and the role of the echographic examination in such cases.

## 1. Introduction

Liquid silicone for intravitreal injection as an adjunct in the treatment of complicated retinal detachments was first described 50 years ago [1]. Since then, there have been several reports on the anterior and posterior segment complications due to silicone oil, such as its emulsification, cataract formation, keratopathy, ocular hypertension, glaucoma, retinal toxicity [2, 3], and migration from vitreous cavity [4, 5]. Free silicone oil within tissues can cause a chronic granulomatous inflammatory reaction, which, depending on the location, can result in impairment of involved structures [4, 6].

Among others, a migration of silicone oil in the subconjunctival space as a rare complication has been reported [5].

We report here the echographic features of one case of a large subconjunctival cyst following silicone oil injection as internal tamponade with the purpose of showing how, sometimes, ugly pictures can be very useful in making a differential diagnosis. This article in addition provides a possible explanation of such cystic formation and discusses the risk factors and the role of echographic examination in such cases.

## 2. Case Presentation

A 39-year-old white female complaining of the presence of conjunctival hyperemia and tearing in the right eye (RE) was sent to us for an outpatient surgical consultation.

The RE visual acuity was hand motion; the intraocular pressure was 14 mmHg. The ocular examination revealed the presence of a mild ptosis, ocular inflammation, and cataract, which did not allow the visualization of the posterior segment.

Her history revealed she had undergone vitreoretinal surgery for retinal detachment few months earlier.

**Figure 1:**
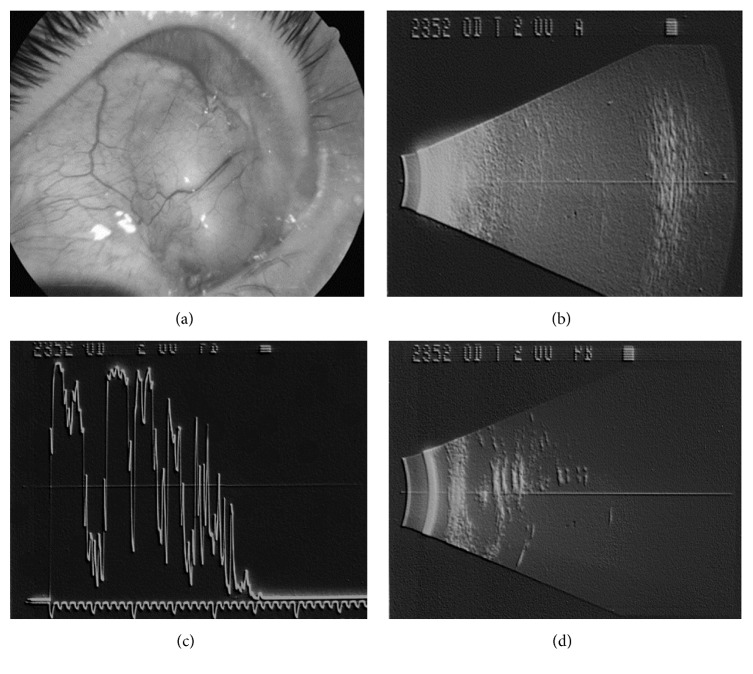
(a) Picture of the dome shaped lesion in the upper nasal quadrant. (b) B scan echographic image showing a classical lengthened image with a flattening and weakening of the posterior eye wall, for the presence of silicon oil in the vitreous chamber. (c) A scan echographic image showing a medium-low reflective lesion followed by a chain of multiple signals. (d) B scan echographic image showing a classical lengthened and weakened image due to the presence of silicon oil in the upper medial episcleral space.

In the upper nasal quadrant a dome shaped lesion was detected, which arose with a differential diagnosis with scleral buckling extrusion, tumor, dermoid, ocular cysticercosis, and abscess (Figure 1(a)). The clinical diagnosis was suspected scleral buckling extrusion but, due to the lens opacities, the patient underwent an echographic examination to detect the posterior pole integrity. The echographic examination showed very poor pictures of the posterior eye wall for a typical shadowing, diagnostic for the presence of silicone oil in the vitreous chamber (Figure 1(b)), and demonstrated a cystic lesion, in the upper medial episcleral space (Figures 1(c) and 1(d)) that had the same echographic features as the silicone oil in the vitreous chamber, with a medium-low reflectivity followed by a chain of multiple signals due to the so-called surface waves, which do not occur in soft tissues but could appear in spherical foreign body-like structures.

Therefore the echographic diagnosis was presence of silicone oil in the subconjunctival space.

After the explanation of the purpose of the surgery, a written informed consent was obtained from the patient, and she was sent to the surgical theatre to remove the cystic lesion. During the conjunctival opening a leak of silicon oil was observed and visualization of a stitch on malacic sclera could be revealed.

After the complete removal of the extruded silicone oil, the wound was closed with a conjunctival sliding flap.

## 3. Discussion

The silicon oil in the vitreous chamber gives a peculiar feature, because it has an absorbing effect and slows the speed of the ultrasounds, “weakening” and flattening the structures behind it.

This “ugly” picture allowed us to detect the presence of silicon oil in the subconjunctival space.

The presence of extraocular silicone after endocular surgery is a rare occurrence and few cases of migration out of the eye have been reported.

Silicon oil migration into the brain, and more specifically in the cerebral intraventricular system [7], in the suprachoroidal space [8, 9], and in the subconjuntival space through glaucoma drainage systems implants, has been described.

To the best of our knowledge, the presence of silicone oil in the subconjuntival space associated with proptosis and ocular inflammation, in a patient with history of ocular trauma and retinal surgery for retinal detachment, has been described in only one report [3]. In that case the complication appeared 13 years after surgery, whereas in our patient only few months had passed from the vitreoretinal surgery.

Another paper presented five cases of subconjunctival cysts following silicone oil injection: the interval between silicone oil injection and subconjunctival cyst diagnosis varied from 15 days to 27 months [5]. In one case the subconjunctival cyst appeared after Molteno implant; in two other cases it was seen after multiple surgeries. All the described cases received silicon oil injection and scleral buckling, in combination or in different surgical times.

Our patient was quite different because no multiple surgeries were performed and no glaucoma drainage system had been implanted. Moreover it is the first where the echographic characteristics of a silicon oil cyst have been described.

Several mechanisms by which the silicone could migrate from vitreous cavity to subconjunctival space have been reported [5, 10]. This migration can occur either during or after surgery.

During surgery, some oil particles can be trapped in the orbital spaces during injection, particularly when silicon oil tamponade is combined with scleral buckling procedures. However, if the sclerotomies are achieved with limited conjunctival opening, and profuse irrigation with the balance salt solution is performed before closing the conjunctiva, the chances of experiencing this complication could be reduced [5].

Postoperatively, leakage of silicone oil can occur under two different circumstances:

1. In case of improper sclerotomy's closure, specially following multiple surgeries, or in high myopic-staphylomatous eyes with thin sclera, sutures tend to cut through the tissues leaving large suture tracks.
2. An intraocular pressure increase (e.g., in the presence of secondary glaucoma) causing a wound dehiscence could be the other postoperative cause of subconjunctival collection of silicone oil [5].

Moreover, if the subconjunctival migration is associated with choroidal detachment, other two mechanisms could be taken into account:

1. An incorrectly placed infusion cannula could start the passage of oil, and coexistent transient hypotony towards the end of the procedure could also cause a local choroidal detachment, allowing the tip of the infusion cannula to enter the suprachoroidal space [10].
2. Another explanation could be an epiretinal traction, allowing a separation between the choroid and the sclera, thus promoting egress of silicone oil into the suprachoroidal space [10].

The presence of silicone oil in the subconjuntival space could be then explained by a subconnective progression through the malacic sclera, the latter deriving from the silicon oil toxicity. Another concomitant factor promoting oil leakage could have been postoperative hypertonia.

In our case choroidal detachment was not present; furthermore we were not aware of the patient's surgical procedure and postoperative data, such as ocular tonometry and retinal features.

This case report shows the role of the echographic examination in the diagnosis of silicon oil leakage, whereas in the other cases, the diagnosis required the use of computerized tomography and biopsy of the subconjunctival mass.

In conclusion, our report underlines the role of ocular echography to correctly diagnose such a rare complication of retinal surgery and the importance of frequent follow-up, especially in the first months after surgery.
